# Correction: Corrigendum: Magnetic Tunnel Junction Mimics Stochastic Cortical Spiking Neurons

**DOI:** 10.1038/srep46894

**Published:** 2017-08-29

**Authors:** Abhronil Sengupta, Priyadarshini Panda, Parami Wijesinghe, Yusung Kim, Kaushik Roy

**Keywords:** Electrical and electronic engineering, Magnetic devices

10.1038/srep30039

This Article contains an error in Equation 2.

should read:

